# Analysis of Treatment of Erysipelas

**Published:** 1854-02

**Authors:** Sanford B. Hunt


### ECLECTIC DEPARTMENT.

### *The Traetment of Erysipelas Analyzed.*

BY SANFORD B. HUNT, M. D.

I propose to bring the various remedies employed in the treatment of erysipelas to the test of a critical analysis, based on the ascertained facts of the disease; laying down first the proposition, that we should exhibit no remedies without knowing why we do so.

When a few years since malignant erysipelas first prevailed to an alarming extent in this section of country, the views of medical men both as to its pathology and tendencies, as to its treatment, were unsettled and unsatisfactory. The epidemic of 1844 and '5, was exceedingly fatal, as might be expected in this state of medical opinion. Sundry points of pathology were at that time settled in the minds of thinking and observing men. Among these the following may be laid down as propositions, then verified and placed among the facts of the profession.

1st. Erysipelas is a contagious exanthem, originating in the presence of a specific blood poison, either conveyed into the system by contagion, or developed there by certain morbid processes not understood.

2nd. The tendency of erysipilas is toward recovery by the self elimination of the blood poison.

3d. The action of this poison depresses the vital powers, but not usually to a fatal degree. When the poison is directed from the surface toward the nervous centers, we shall have symptoms much more alarming than when it expends itself in cutaneous inflammation.

Probably these propositions are not novelties, and will be readily acceded to by all who have carefully watched the disease in question. I shall not, therefore, enforce them by any argument, but shall confine the scope of this article to sundry deductions as to treatment, devised from these data.

1st. If erysipelas is a contagious exanthem, analogy would lead us to suppose that its management should be governed by the same rules which guide us in the other principal exanthemata, viz., scarlatina, rubeola, variola, varicella, and continued fever. All these disorders belong to the same family, and are subject to the same laws. All have their origin in contagion, though some of them may be self-developed. All of them depend upon blood poison as their cause. All are self-limited; in all of them an abortive treatment is perhaps impossible; in all of them the degree of danger depends upon the degree to which the nervous centers are affected by the poison; and in all of them that treatment will be most sucessful which most favors the elimination of the specific poison. These anlaogies are worthy of consideration. We may argue with tolerable certainty from one to the other, and may readily conclude that one principle of treatment should govern the whole. And in the whole art of therapeutics no principle is better established than that depletion is inadmissible in all this class. Erysipelas is a blood poison. Therefore any merely local treatment must be generally insufficient. I say *generally*, because there are many cases in which this poison expends itself almost as locally as does vaccinia in its usual mild career. Of coure, in such cases local treatment will be not only sufficient but superfluous. Prof Bennett saw in the Hotel Dieu, a number of cases which were receiving no treatment—M. Louis asserting that erysipelas of the scalp was never fatal. And this accords with all our preconceived notions of eruptive disease. When the eruption comes out fair, thus affording the best chance for elimination, when it occupies only its natural locality, the skin, and is thus uncomplicated with visceral inflammation, we expect a recovery *tuto, citoque, jucunde,* without much, if any medical interference, and the only judicious treatment in any of these diseases, is that which directs the morbid matter in common with the whole tide of circulation toward the surface.

The elimination of the specific poison of erysipelas is conducted by the usual emunctories—the skin and the kidneys. Free diaphoresis exerts a most favorable influence upon the progress of the disease, tending to shorten the process of elemination. The occurrence of desquamination, even when no bullæ exist, indicates the effusion of fluid beneath the cuticle, and it is not improbable that this effusion subtracts its quantum from the sum total of disease. The relative quantity of urea in the blood seems to have in this, as in all other exanthemata, an influence upon the severity of the disease. But what this influence is we do not know, though we may hope that with our present means of investigation, the thing may soon become clear.

In erysipelas, as in other desquamative disease, we find frequently —not always—the accompanying sign of albuminuria. This occurs at the period of declination, and depends upon the solution of the renal epithelium in the urine. That this is a part of the process of elimination is not proven, and it may be found to depend upon a local disorder of the kidneys, incident to the increased secretion of urea occurring at the period of declination. The plain indications of treatment derived from these natural phenomena, are to encourage the secretions of both the skin and the kidneys.

2d. The tendency of erysipelas is toward recovery. This is prov en by the small average of deaths. The recovery of very many cases in which the disease has run its course uninfluenced by medication, and finally by the analogy derived from the natural history of other eruptive diseases.

3d. Whenever the symptoms become alarming it is not from the acute or destructive character of the inflammation, but from a depression of the nervous energy. Grangrene is of extremely rare occurrence. Abscess is more common, but still so unusual as not to be classed among the natural sequelæ of the disease. When erysipelas assumes a bad type, we find the inflamed surface loses its lively red, and acquires a dark, purple hue; the skin is dry; the urine suppressed; the tongue has a dry brown crust; the teeth are covered with sordes; the pulse grows frequent and fluttering; delerium and coma are present; in a word we have typhoid symptoms. All this retinue of bad signs have their origin in a morbid impression on the great nervous centers, and we readily draw the indication of a supporting treatment. If the disease pursues its natural course, finding outlet by the skin and kidneys, we shall have the vital powers unimpaired, the reson clear, and the pulse—the great indicator of nervous disorder—undisturbed. But when from any cause the poison is retained in the circulation, and goes on increasing by zymosis, we shall find that the brain becomes congested—that the disease has left its wonted channels, and is expending itself upon the organs more important—upon the great moving power—the *nodus vitœ* itself.

It seems to me that if what I have advanced be true, we have a sufficient basis for scientific and successful treatment of erysipelas, which by a parity of reasoning will apply to the other eruptive zymotics. It is not probable that those things yet unexplained will have the same important bearing upon treatment as the facts already in our possession.

*Treatment.* Most of our systematic authors speak of bleeding as admissible, and even praiseworthy, in the country, but not admissible in the form of the disease seen in cities. But if what has been said about the tendencies of the disease to tyhpoid action, the little danger of unfavorable result from the extent of violence of inflammatory action, and the importance of maintaining the nervous system in full vigor be of any moment, then is a bleeding in erysipelas as unphilosophical as in continued fever, variola, or any other exanthem. The safety of the patient lies in the activity and lively character of the local inflammation; for the extent of the inflamed surface is only an indication of the amount of blood poison and the activity of the zymosis.

The diaphoretic treatment seems, at first view, to be theoretically and practically correct, as indeed it is. But to secure diaphoresis, we should not administer drugs which, by their depressing influences, lessen the nervous energies—*e. g.*, antimony. There are, however, diaphoretics of a different class to which no such objections can apply as the acetate, and carbonate of ammonia. Whisky punch, which at first view would seem a good stimulant diaphoretic, is objectionable on account of the reaction and torpor of the nervous system following its use.

Diuretics are also indicated, but the antagonism of the skin and kidneys renders it difficult to meet both these indications at once. Perhaps colchicum, tending directly to the removal of urea, might be found the best of diuretics.

But even these indications, important as they are, yield to the necessity, in serious cases, of supporting the nervous powers, Quinine is thus valuable, and here do we find what I conceive to be the true theory of Hamilton Bell's treatment by the Tinct. Ferri Muriatis. He himself argues that the capillaries are in an atonic state, and that the system being rapidly saturated by this powerful astringent, the atony is removed. Evidently if this were untrue, cold affusion would be equally successful. In the only case in which I have had the opportunity to observe the action of this remedy, it was commenced 12 hours after the appearance of the eruption. The pulse was 100, full, and firm. Fifteen drops of the muriated tinct, were given every two hours. A cathartic was given with the first dose. The patient was a blacksmith, aged 35. There was no other treatment, either general or local. Under the remedy he sweat profusely, and forty-eight ho urs after the treatment commenced, the pulse was 72 and soft, and the swelling, which had occupied the whole face above the mouth, closing both eyes, was declining. He convalesced rapidly. This is a single case, Perhaps a collection of cases might give a different result, though this case is similar to those reported by Dr. Bell in all particulars. In attempting to explain the action of the remedy wemay say this much: The iron is a good tonic, and is *prima facie*, as well adapted to the disease as quinine. Perhaps by the action of the iron, the nervous system is exalted to a pitch which enables it to exert its whole energies in throwing off the poison.

*Cathartics.* Probably there is no case of erysipelas which will not at some time in its course, be the better for cathartic action.

Another corollary to our proposition, is that all local applications further than those tending to relieve itching and pain, are unnecessary and mischievous, If by local applications you cut short the inflammation, you lengthen correspondingly the disease. This may not hold true in a far advanced stage of the disease.

It will be seen that by this process of analysis from established facts, we rather subtract from, than add to our armamentaria against this disease. The whole treatment is reduced to a simple combination of tonics, diaphoretics, and diuretics. The peculiar remedies which have my preference I have indicated, viz: iron, colchicum, and the acetate of ammonia; but it is probable the other articles of the materi medica may fulfill the same indications, nearly, if not equally as well.—*Buffalo Med. Jour.*

---

*Stranguary from Blisters.*—Dr. Anderson of Alabama, believes that stranguary can uniformily be prevented "by smearing the plaster with oil of turpentine" before applying it.

